# Self-referred patients at the Emergency Department: patient characteristics, motivations, and willingness to make a copayment

**DOI:** 10.1186/s12245-014-0030-7

**Published:** 2014-07-22

**Authors:** Janneke de Valk, Elisabeth M Taal, Mariette S Nijhoff, Maren H Harms, Esther MM Lieshout, Peter Patka, Pleunie PM Rood

**Affiliations:** 1 Department of Emergency Medicine, Erasmus MC, Erasmus University Medical Center Rotterdam, Rotterdam, 3000, CA, The Netherlands; 2 Trauma Research Unit, Department of Surgery, Erasmus MC, Erasmus University Medical Center Rotterdam, Rotterdam, 3000, CA, The Netherlands

**Keywords:** Self-referred patients, Emergency Department, General practitioner, General Practitioner cooperative, Copayment, Patient motivations, Health services accessibility

## Abstract

**Background:**

In many countries, including the Netherlands, a substantial number of patients visit the Emergency Department (ED) without a referral by a general practitioner. The goal of this study was to determine the characteristics and motivations of self-referred patients (SRPs) at the ED. The secondary objective was to explore SRPs’ opinion about copayments.

**Methods:**

A survey, in seven different languages was performed among SRPs from October 2011 until January 2012 at an academic ED in the Netherlands. Patients were included on 21 day-, 21 evening-, and 21 nightshifts during week and weekend days equally. Patient characteristics, motivations, complaints, diagnosis, and the opinion regarding copayments were examined.

**Results:**

A total of 436 SRPs were included (response rate 82%). Forty-seven percent of the ED population was self-referred. SRPs were mainly male (58%), between 18 and 35 years (54%), Dutch (67%), single without children (42%), and low-educated (73%). The most commonly presented complaints were of musculoskeletal origin (35%). Expected need for additional medical care (e.g., X-rays, blood tests) was the reason to visit the ED for 28% of the SRPs. Around 30% of the SRPs were not prepared to pay for an ED visit. Fifty percent of SRPs were prepared to pay up to 25 or 50 EUR. Highly educated patients were willing to pay more than patients with a low level of education (*p* < 0.05).

**Conclusions:**

SRPs (47% of the total ED population) are often young men with musculoskeletal complaints. They are convinced that additional medical tests are necessary. About 70% of the SRPs are willing to make a copayment, half of the SRPs with a maximum between 25 EUR and 50 EUR. As highly educated SRPs are prepared to pay more, introducing copayments might influence equity in health care accessibility.

## Background

Annually, approximately 2 million patients present themselves to an Emergency Department (ED) in the Netherlands [[1]]. Thirty percent of them visit the ED without being referred by a general practitioner (GP) [[2]].

In the year 2000, after-hours GP practices, ‘GP-cooperatives’, were established in the Netherlands. Patients with health problems can either visit their GP during office hours or a GP-cooperative for after-office-hours health care. The GP functions as a gatekeeper for in-hospital care and can refer patients to the ED or hospital medical specialties. However, medical care at the ED is also provided to patients without a GPs referral to the ED.

Several studies exploring the characteristics and motives of self-referred patients (SRPs) for their visits to the ED have been carried out [[3]–[8]]. However, most of them were conducted before or during the introduction of the GP-cooperatives [[3],[6]–[8]]. Furthermore, some studies investigated the presentation of SRPs to the ED only during out of office hours for regular medical care or were focused on specific patient groups, for example, surgical patients [[6]].

Since a visit to the ED is nearly three times more expensive (for the health care system) than a visit to a GP-cooperative and even five times more expensive than a visit to the patients’ own GP, it is believed that substantial savings could be made by reducing the number of SRPs to the ED [[9],[10]]. A potential solution to this problem could be the introduction of copayments for SRPs for their visits to the ED [[11]]. In some countries such as the USA, it is not uncommon for patients to make a copayment. Yet, in other countries, including the UK and the Netherlands, copayments are quite unusual [[12]]. Patients’ attitude towards copayments in the ED has not been studied before in a representative patient population.

Access to health care and health care costs of SRPs in the ED remain important social and political topics. We therefore aimed to determine the current characteristics and motivations of SRPs visiting the ED. The secondary objective was to explore SRPs’ opinion towards copayments.

## Methods

This cross-sectional study was carried out at the ED of a tertiary university hospital in Rotterdam, the Netherlands. This ED has an average case load of 75 patients per day, about 25,000 patients a year, of which approximately 50% is self-referred.

All patients visiting the ED without a referral from a physician or ambulance personnel, aged 18 years or older, were eligible for participation in the study. Patients were excluded if they were unable to fill out the questionnaire, were already participating in another ED study, or if no informed consent was obtained. For SRPs who presented themselves more than once to the ED during the study period, only the first visit was included.

The study was performed by dedicated researchers from October 2011 until January 2012. They registered all consecutive patients during week days and weekend days equally, on 21 day-, 21 evening-, and 21 nightshifts that were randomly selected. Patients who met the inclusion criteria and none of the exclusion criteria were asked to fill out a short questionnaire.

A short questionnaire about patients’ characteristics (age, gender, city of residence, civil status, level of education: highest level of education in categories ‘high’, college; ‘intermediate’, high school; ‘low’, primary school or vocational training), reasons, and motivations of SRPs to visit the ED was developed. Furthermore, patients’ opinions on paying for being seen at the ED were asked. To improve face and content validity, the questionnaire was tested on 26 medical professionals and 26 volunteers. The final questionnaire was translated and made available in six commonly spoken foreign languages in The Netherlands: English, French, German, Spanish, Polish, and Turkish.

Besides the questionnaire, the following information from the hospital information system was obtained: test results, including laboratory data, X-rays, CT scans, and EKGs; history of medical complaints; diagnosis and treatment.

Statistical analyses were performed using SPSS (Statistical Package for the Social Sciences, IBM, NY, USA) software version 17. Associations between categorical variables were assessed with Pearson’s chi-square tests. A *p* value <0.05 was taken as threshold of statistical significance.

The study was conducted according to the principles of the Declaration of Helsinki (October 2008) [[13]] and institutional approval was obtained from the research ethics board of Erasmus MC Rotterdam, prior to the initiation of the study.

## Results

**Figure 1:**
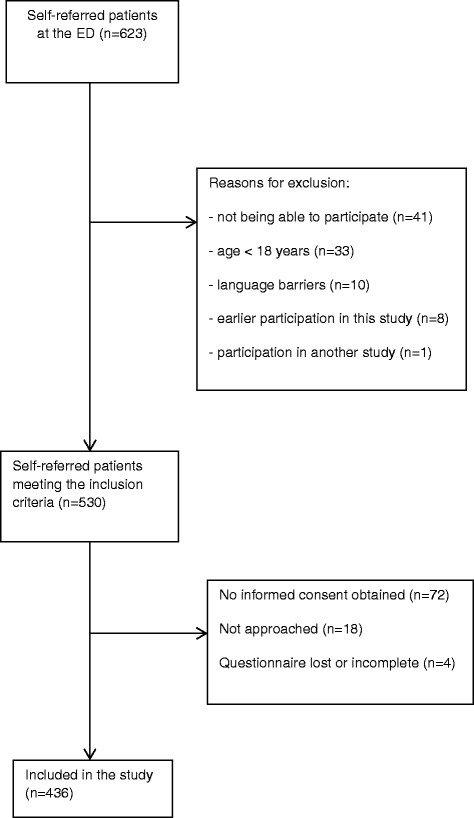
Study flowchart.

During the study period, a total of 1,315 patients presented to the ED of whom 623 (47%) were self-referred. Five hundred thirty patients met the criteria for inclusion, of whom 72 refused informed consent; 18 patients were not approached, and in four cases the questionnaire was lost or incomplete (Figure 1). A total of 436 questionnaires were available for analysis (overall response rate of 82%), 87% (*n* = 381) were in Dutch, 7% (*n* = 29) in English, and 6% (*n* = 26) in one of the five other languages.

SRPs were mainly male, aged 18 to 35 years, Dutch, single without children, and had a low or intermediate level of education (Table 1). More than 80% of all SRPs were familiar with GP-cooperatives. However, Dutch citizens were significantly more aware of this concept compared to other nationalities (93% versus 60%, *p* < 0.01).

**Table 1:** **Characteristics of the self-referred patients (*n*** **= 436)**

		**Percentage**	**Number**	***p*** **value**^**a**^
Gender	Male	58	251	<0.01
	Female	42	185	
Age categories (years)	18 to 35	54	234	<0.01
	35 to 65	40	173	
	65+	7	29	
Residence	Inner city	70	305	<0.01
	Urban area	21	90	
	Netherlands, outside urban study area	6	27	
	Abroad	3	14	
Nationality	Dutch	67	290	<0.01
	Other	33	146	
Level of education	Low or intermediate	73	315	<0.01
	High	27	116	
Civil status	Single, no children	42	182	<0.01
	Partner with children	24	105	
	Single with children	18	77	
	Partner, no children	12	53	
	Other	4	17	
Shift	Day	45	198	<0.01
	Evening	35	153	
	Night	20	85	

^a^Chi-square test.

The reasons for SRPs to visit the ED are shown in Table 2. The main reason was the expected need for additional medical tests (e.g., X-rays, blood tests) that could not be performed by the GP. Other important reasons were the accessibility or location of the ED and/or GP-cooperative (there was no GP/GP-cooperative nearby or the location was unknown, ED was nearby). Around 11% of the respondents indicated they were not registered with a GP. The proportion of Dutch people not having a GP was significantly lower than that of non-Dutch people (4% versus 26%, *p* < 0.01).

**Table 2:** Dominant reasons for self-referred patients for visiting the Emergency Department

	**Percentage**	**Number**
Patients’ assumption that medical care was needed that a GP cannot provide (e.g., X-ray, blood tests)	28	120
Patient was already under specialist care at the study hospital	17	74
There was no GP/GP-cooperative nearby	16	69
Patient could get help earlier at the ED	15	63
The ED was located nearby	11	49
Patient was not registered with a GP	11	48
Patient could not reach the GP/GP-cooperative	7	32
The location of the GP-cooperative was unknown	5	23
Previous negative experience with the GP/GP-cooperative	4	18
Patient had no faith/trust in the GP	3	14
On the advice of others	3	13
Patient perceived the complaint was urgent	2	8

Respondents (*n* = 436) could give more than one answer.

The most commonly presented complaints were of musculoskeletal origin (35%) and skin problems (18%).

Of all SRPs, more than half (56%) of them underwent additional medical tests, mainly X-rays or blood tests. For 45% of the patients, some kind of follow-up after the ED visit was arranged, including a consultation with a medical specialist (31%, *n* = 137) or an appointment at the outpatient department (32%, *n* = 138). Seven percent (*n* = 31) of the SRPs were admitted to the hospital.

When asked regarding the amount of copayments, 29 SRPs (7%) were not prepared to answer this question. Around 30% of the SRPs were not willing to pay for visiting the ED, and half of the SRPs were prepared to pay up to 25 or 50 EUR (Table 3). People with a higher level of education were significantly more willing to make a (higher) copayment (15% versus 6%, *p* < 0.05). The willingness to pay more than 100 EUR was significantly higher on the weekend than on weekdays (12% versus 6%, *p* < 0.01).

**Table 3:** Amount of money self-referred patients are willing to pay for visiting the Emergency Department

	**Low/Intermediate level of education (% (*n*** **= 293))**	**High level of education (% (*n*** **= 111))**	**Total (% (*n*** **= 404))**
€ 0	33 (96)	26 (29)	31 (125)
Max € 25	31 (90)	23 (26)	29 (116)
€ 26 to 50	21 (61)	23 (25)	21 (86)
€ 51 to 100	10 (30)	13 (14)	11 (44)
>€ 100	5 (16)	15 (17)	8 (33)

*n* = 404; *p* <0.05; Chi-square test.

## Discussion

This study showed that a substantial part of the visitors of our ED (47%) is self-referred. In our study population, SRPs were most often male young adults, age between 18 and 35 years, and single without children. The important reasons to visit the ED instead of the GP were the patients’ perception of needing medical care that the GP could not provide, the fact that the patient was already under specialist care in the hospital, or the proximity of the ED.

SRPs at the ED have been shown to be predominantly young male patients with musculoskeletal injuries [[4],[14]], a finding that was confirmed in this study. Most of the SRPs in our study had a low or intermediate education level, while other studies found the opposite [[10],[15]]. This difference could be influenced by the level of education of citizens in the city area of our study location, which is lower than in other urban areas in the Netherlands [[16]].

Several studies demonstrated that an important reason for SRPs to visit the ED was the patients’ impression that their medical complaint was urgent, or the opinion that the resources in hospitals are better than in a GP setting [[3],[17],[18]]. The expectation that it would be necessary to use diagnostic facilities was a reason to visit the ED for 22% to 36% of SRPs [[4],[5]]. Other frequently mentioned reasons are the location or easy accessibility of the ED [[18],[19]]. In this study, we confirmed the patient motivations mentioned above. Additionally, in this study, 17% of the SRPs visited the ED because they were already under specialist medical care in the study hospital. This could possibly be explained by the fact that the study hospital is a tertiary university hospital.

While in our study, 45% of the patients needed some kind of medical follow-up; others found that medical follow-up was only necessary in 21% of SRPs [[20]]. The proportion of SRPs that was admitted to the hospital in our study was only 7%, which was still higher than the 4% reported in a study in a comparable inner city hospital [[21]].

One of the reasons to gain insight into the motivations of SRPs to visit the ED is that SRPs (47% of the ED population in our study) may contribute to overcrowding of the ED. Overcrowding can result in delay in diagnosis or treatment and reduced quality of care [[22]]. Others believe overcrowding of EDs is mainly the result of dysfunction of the surrounding health care system [[23]]. Also, since SRPs are composed of a group with relatively less urgent health problems, they may form a relatively constant and inexpensive proportion of the workload at the ED [[24],[25]].

A measure to diminish the number of ED visits might be cost sharing for emergency care. Several studies in the USA demonstrate a significant reduction in ED use varying from 12% to 23% after the introduction of a copayment [[26],[27]]. It is suggested that interventions aimed at ED cost sharing could be effective in reducing ED use [[28]]. In the Netherlands, members of the Dutch Health Care Consumer Panel of the Netherlands Institute for Health Services Research (NIVEL) were questioned about their opinion towards copayments when visiting the ED as a SRP [[29]]. An obligation to pay more than 25 EUR for a presentation at the ED would prevent half of the SRPs from coming to the ED. If a patient had to pay more than 100 EUR, only 15% would still visit the ED. In this study we obtained similar results. We showed that nearly one third of SRPs is not prepared to make extra payments. A payment of more than 25 EUR would cause 60% of SRPs to abandon visiting the ED. Only less than 10% is willing to pay more than 100 EUR, which also depends on the level of education of the SRP.

We can conclude that a payment of more than 25 EUR could potentially reduce the number of SRPs to the ED; however, this may have detrimental side effects. It may be ‘unethical’ to create a financial barrier as most patients cannot judge whether it is necessary to visit the ED [[5],[30]]. Some studies indicate that most SRPs at the ED could be treated by a GP [[15],[31]]. Others state that coming to the ED is often necessary for SRPs because of additional medical tests, treatment, or medical follow-up the GP could not provide [[32]]. In our study this was the case in 56% of the SRPs.

Furthermore, introducing a copayment may influence the accessibility of the health care system and cause inequity in health, as it is shown by our study that higher-educated respondents are more willing to pay a minimum of 100 EUR for their visit. It is likely that these SRPs have a higher income, making it easier for them to pay extra.

### Strengths and weaknesses

The response rate of this study was high (82%). The questionnaire used had been translated into several languages, aiming to limit potential for selection bias. Furthermore, our study has a high internal validity as it was carried out by means of a questionnaire at the moment the respondent is visiting the ED and not by consulting a health care consumer panel [[5],[29]]. The questionnaire was tested on face and content validity.

This study had some limitations. The study was carried out in one university hospital without a GP-cooperative on site. This situation may, in general, not be representative for all other hospitals. However, as the study was performed in an inner city hospital with a large proportion of SRPs, the results can make a meaningful contribution to the literature.

## Conclusions

SRPs visiting the ED are relatively often young men with musculoskeletal complaints. The most important reason to visit the ED is the patients’ expectation of the need of additional medical tests. In our population, almost 70% of the SRPs are willing to pay an additional fee to visit the ED, mostly with a maximum of 25 EUR (29%) or between 26 EUR and 50 EUR (21%). Eight percent of the SRPs are willing to pay more than 100 EUR. As highly educated SRPs are prepared to pay more, introducing copayments might influence equity in health care accessibility.

## Abbreviations

ED: Emergency Department

GP: eneral practitioner

SRP: self-referred patient

## Competing interests

The authors declare that they have no competing interests.

## Authors’ contributions

JV has made substantial contributions to the design of the study, supervising the data collection, and the writing of the manuscript. ET has made a substantial contribution to the analysis and interpretation of data and the writing of the manuscript. MN and MH have made substantial contributions to the data collection, analysis, and interpretation of the data. EL has been involved in the methodological design of the study and has supervised the analysis of the data and revising the manuscript. PP has been involved in supervision of all phases of the study including revision of the manuscript. PR has made substantial contributions to the design of the study, the supervision of data collection, the interpretation of data, and the writing of the manuscript. All authors read and approved the final manuscript.
