# Erratum: Self-referred patients at the Emergency Department: patient characteristics, motivations, and willingness to make a copayment

**DOI:** 10.1186/s12245-015-0058-3

**Published:** 2015-05-09

**Authors:** Janneke de Valk, Elisabeth M Taal, Mariette S Nijhoff, Maren H Harms, Esther MM Van Lieshout, Peter Patka, Pleunie PM Rood

**Affiliations:** Department of Emergency Medicine, Erasmus MC, Erasmus University Medical Center Rotterdam, Rotterdam, 3000 CA The Netherlands; Trauma Research Unit, Department of Surgery, Erasmus MC, Erasmus University Medical Center Rotterdam, Rotterdam, 3000 CA The Netherlands

## Erratum

In the original version of this article [1], the author name ‘Esther MM Van Lieshout’ was incorrectly listed as ‘Esther MM Lieshout’.
